# Alcohol Metabolism into Acetaldehyde in Developing Cerebral Arteries

**DOI:** 10.3390/ijms27146463

**Published:** 2026-07-21

**Authors:** Rika M. Morales, Shiwani Thapa, Anna N. Bukiya

**Affiliations:** Department of Pharmacology, Addiction Science and Toxicology, College of Medicine, The University of Tennessee Health Science Center, Memphis, TN 38103, USA

**Keywords:** enzyme activity, early development, prenatal alcohol

## Abstract

Alcohol exposure during pregnancy leads to fetal alcohol spectrum disorders (FASD), yet the mechanisms through which alcohol disrupts the developing cerebrovasculature remain poorly defined. Acetaldehyde, the first oxidative metabolite of alcohol, can alter vascular function, but whether developing cerebral arteries possess intrinsic capacity to generate acetaldehyde is unknown. Alcohol is primarily oxidized by alcohol dehydrogenase (ADH), cytochrome P450 2E1 (CYP2E1), and catalase (CAT), and their local metabolic activity may contribute to cerebrovascular vulnerability. In this study, cerebral arteries were isolated from postnatal day (PND) 10 C57BL/6J mouse offspring (third trimester-equivalent to human pregnancy), and incubated ex vivo with physiologically relevant alcohol concentrations (13 or 50 mM). Acetaldehyde generation, transcript expression, protein abundance, and catalase-dependent metabolism were evaluated. Alcohol exposure produced a concentration-dependent increase in acetaldehyde generation within developing cerebral arteries, with comparable responses between males and females. Transcript analysis revealed that *Adh1*, *Cyp2e1*, and *Cat* were expressed across developing tissues; however, Western blotting showed that catalase was the only alcohol-metabolizing enzyme detectable at the protein level within developing cerebral arteries. Accordingly, catalase inhibition by sodium azide altered acetaldehyde production, revealing a significant blocker–sex interaction at the higher inhibitor concentration (0.06 mM). In summary, our ex vivo findings demonstrate that developing cerebral arteries possess intrinsic metabolic capacity to oxidize alcohol to acetaldehyde and that catalase plays an essential role in supporting this process at this developmental stage. These results point to a previously unrecognized metabolic pathway within the developing cerebrovasculature that may potentially contribute to early-life vulnerability to alcohol exposure.

## 1. Introduction

Developmental exposure to alcohol gives rise to fetal alcohol spectrum disorders (FASD), a group of lifelong conditions characterized not only by structural and functional abnormalities in the central neurons and glia but also by significant cerebrovascular dysfunction [1,2,3,4]. FASD affects an estimated 7.7 newborns per 1000 live births worldwide, with some subpopulations showing rates 10–40 times higher [5]. Prenatal alcohol exposure (PAE)—the defining criterion for FASD—disrupts cognitive, physical, and behavioral development [2,4] and remains the leading preventable cause of neurodevelopmental disability globally [5,6,7,8]. Despite its substantial public health and economic burden, no treatments reverse the long-term consequences of PAE, underscoring the need to identify underlying mechanisms and therapeutic targets that reduce early-life vulnerability to alcohol exposure.

Although PAE affects multiple fetal organs and systems, the developing brain is uniquely sensitive, exhibiting reduced volume and persistent structural and functional deficits [9,10,11,12]. Proper cerebral circulation is essential for brain development, supporting neural progenitor migration, oxygen and nutrient delivery, and waste clearance [13,14,15,16,17]. Cerebral arteries (anterior, middle, posterior, and basilar) mature rapidly beginning at around 24 days of human gestation, with the Circle of Willis being largely established by the end of the first trimester [16,18]. However, this early period primarily reflects morphological establishment of the cerebrovascular network rather than functional maturation [16,19]. Cerebrovascular development continues throughout later gestation and during the third trimester, cerebral arteries develop increasingly mature contractile and metabolic processes [20,21]. This period is therefore characterized by functional refinement of cerebrovascular regulation [19]. By the end of the third trimester, cerebral arteries establish autoregulation to further support brain growth and rising metabolic demand [21]. Thus, cerebral blood vessels play key roles in supplying blood to migrating neurons and supporting formation of the microvasculature [17,22]. Perturbation during these developmental windows can produce long-lasting deficits, yet the mechanisms by which alcohol directly disrupts cerebrovascular maturation remain poorly understood. Local alcohol metabolism is one potential mechanism of alcohol toxicity, as alcohol’s reactive metabolite acetaldehyde can modulate vascular tone, induce oxidative stress, and impair arterial cell function [23,24,25,26].

Alcohol can be oxidized to acetaldehyde through three pathways: by alcohol dehydrogenase (ADH), cytochrome P450 2E1 (CYP2E1), and catalase (CAT) [27,28]. While these enzymes are well characterized in the adult tissues, and both CYP2E1 and CAT have been reported in developing brain regions [27,28,29,30,31], it is unknown whether developing cerebral arteries express any of these enzymes or possess intrinsic capacity to generate acetaldehyde. In this study, we use a mouse model to investigate whether developing cerebral arteries metabolize alcohol into acetaldehyde at the end of the third trimester-equivalent of human pregnancy using an ex vivo alcohol incubation and enzyme detection approaches. The third trimester was selected because it represents a critical period of functional cerebrovascular maturation characterized by rapid brain growth and emergence of mature vascular reactivity [20,21]. Additionally, this period is marked by a heightened vulnerability to PAE-induced growth, motor, and cognitive deficiencies [32]. Our ex vivo findings demonstrate that developing cerebral arteries may be capable of metabolizing alcohol into acetaldehyde, express alcohol-metabolizing enzymes, and have functional catalase. Our work is performed with alcohol levels (13 and 50 mM) that are reached within blood circulation during mild versus moderate-to-heavy alcohol consumption. Our findings pave the way to addressing a key gap in understanding alcohol pathophysiology within the developing cerebrovasculature and raise a possibility that acetaldehyde contributes to pathological effects of early-life alcohol exposure in the brain.

## 2. Results

### 2.1. Developing Cerebral Arteries Produce Acetaldehyde in Response to Alcohol Incubation Ex Vivo

**Figure 1:**
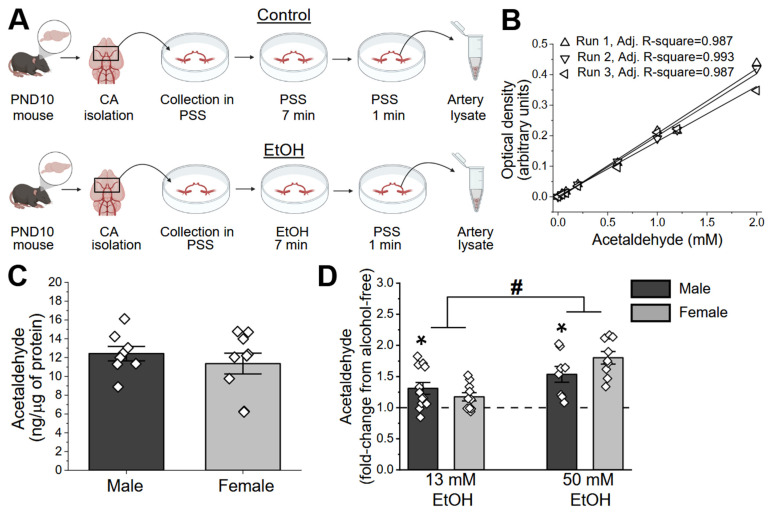
Exposure of isolated cerebral arteries to alcohol ex vivo results in rising acetaldehyde levels. (**A**) Experimental design: Isolated cerebral arteries from PND 10 mouse offspring were first incubated in PSS, then incubated for 7 min in either fresh PSS (control group) or alcohol (EtOH; 13 or 50 mM) solution. Following incubation, arteries were briefly dipped in PSS to remove excess alcohol and immediately lysed for acetaldehyde quantification. (**B**) Calibration plots from acetaldehyde detection assay show linearity within a wide range of acetaldehyde concentrations and reproducibility. Individual fits were obtained using Origin 2023 software (OriginLab Corp 2023, Northampton, MA, USA) from standards of assays that were run on three independent experimental occasions. Adj.: adjusted. (**C**) Baseline acetaldehyde levels obtained from cerebral arteries that were not treated with alcohol. Males: *n* = 8 dams. Females: *n* = 9 dams. *p* = 0.8184 by 2-tailed Mann–Whitney U test. (**D**) Acetaldehyde levels (expressed as fold-change relative to PSS-incubated alcohol-free controls) in developing cerebral arteries from male pups (dark grey) and female pups (light grey) following incubation with 13 mM or 50 mM alcohol. Each point represents data collected from a pooled artery sample from three pups of the same sex from a single dam (male 13 mM EtOH *n* = 12 dams, male 50 mM EtOH *n* = 8 dams; female 13 mM EtOH *n* = 11 dams, female 50 mM EtOH *n* = 9 dams). Control (PSS-incubated) values used for normalization were obtained from male (*n* = 8 dams) and female (*n* = 9 dams) pooled artery samples and are shown in (**C**). Data are presented as mean ± SEM. Outliers were removed prior to statistical analysis (male 50 mM EtOH *n* = 2 dams; female 13 mM EtOH *n* = 2 dams). # *p* < 0.0001 by two-way ANOVA (main effect of alcohol concentration). * *p* < 0.05 by Mann–Whitney U test (significant difference compared to baseline levels).

We first investigated whether developing cerebral arteries can metabolize alcohol through the production of acetaldehyde, the initial metabolite of alcohol oxidation. Cerebral arteries were dissected out from male and female neonatal pup brains (PND 10 ± 1 day; that is the end of third trimester-equivalent of human pregnancy) [33] and immediately placed into physiological saline solution (PSS). Arteries were incubated ex vivo for 7 min with either PSS (control) or physiologically relevant concentrations of alcohol (13 and 50 mM; Figure 1A). Following incubation, arteries were dipped into PSS, lysed, and acetaldehyde content was quantified using a commercially available acetaldehyde detection kit as described in Methods. Calibration plots showed great linearity and reproducibility (Figure 1B). Namely, linear fits of standards within a wide concentration range showed excellent adjusted R-squares (between 0.987 and 0.993). Measurements for these fits were obtained in parallel with the acetaldehyde assessment in the artery samples on each day of the experiment. Spiking artery lysates with a given acetaldehyde level and comparing assay readings with physiologic saline-based standards of acetaldehyde resulted in 91% recovery of the signal in lysate-containing samples. Thus, there was little interference from cerebral artery lysate components with the assay ingredients.

Baseline acetaldehyde levels in alcohol-free (untreated) samples of cerebral arteries were 1.24 × 10^−5^ ± 7.60 × 10^−7^ mg/µg of total protein in males; 1.14 × 10^−5^ ± 1.01 × 10^−6^ mg/µg of total protein in females (Figure 1C). There was no statistically significant difference between baseline acetaldehyde levels in arteries from males versus females (*p* = 0.8184). These baseline levels obtained from alcohol-free (untreated) samples were used to normalize acetaldehyde levels detected in alcohol-treated samples. Alcohol incubation produced a concentration-dependent increase in acetaldehyde levels (Figure 1D). In males, 13 mM alcohol produced a modest elevation in acetaldehyde (1.31 ± 0.09-fold), while 50 mM alcohol elicited a larger increase (1.76 ± 0.39-fold; Figure 1D dark grey). A similar pattern was observed in females, with 13 mM and 50 mM alcohol increasing acetaldehyde to 1.39 ± 0.17-fold and 1.80 ± 0.10-fold above baseline, respectively (Figure 1D light grey).

A two-way ANOVA (factors: alcohol concentration and sex) revealed a significant main effect of alcohol concentration (F(1, 35) = 19.12, *p* < 0.0001), indicating that higher alcohol concentration produced higher elevation in acetaldehyde production within developing cerebral arteries (Figure 1D). There was no significant main effect of sex (F(1,35) = 0.53, *p* = 0.47), and the interaction between alcohol concentration and sex did not reach significance (F(1,35) = 3.81, *p* = 0.059). These findings indicate that male- and female-developing cerebral arteries respond similarly to alcohol incubation. Overall, these results demonstrate that developing cerebral arteries are capable of metabolizing alcohol to acetaldehyde in a concentration-dependent manner, disregarding progeny sex.

### 2.2. Developing Tissues Transcribe Genes That Encode All Three Alcohol-Metabolizing Enzymes

**Figure 2:**
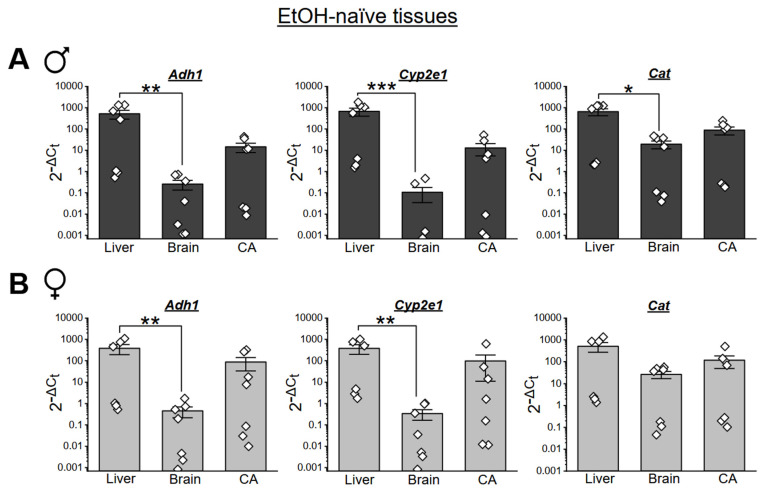
Validation of transcript presence from genes encoding alcohol-metabolizing enzymes in alcohol-naïve developing tissues. Expression of genes that encode alcohol-metabolizing enzymes was confirmed by qPCR analysis of the *Adh1*, *Cyp2e1*, and *Cat* gene transcripts in alcohol-naïve developing liver, brain, and cerebral artery tissue samples. All samples were collected from PND 10 mouse offspring. In the bar graphs, the y-axis represents 2^−ΔCt^ on a logarithmic scale to improve visualization of low-abundance transcripts. Ct indicates the cycle number at which the fluorescence surpasses the threshold to detect the target gene. *Adh1*, *Cyp2e1*, and *Cat* gene expression levels in each sample were normalized to *Gapdh* coding transcript. (**A**) Relative mRNA expression of *Adh1*, *Cyp2e1*, and *Cat* in developing liver (*n* = 7 pups from no less than 3 dams), brain (*n* = 7 pups from no less than 3 dams), and cerebral arteries (n = 7 dams) of male pups. (**B**) Corresponding mRNA expression levels in liver (*Adh1 n* = 7; *Cyp2e1 n* = 7; *Cat n* = 6), brain (*Adh1 n* = 7; *Cyp2e1 n* = 7; *Cat n* = 7), and cerebral arteries (*Adh1 n* = 7; *Cyp2e1 n* = 6; *Cat n* = 6) of female pups. For liver and brain, each data point represents an individual pup; for cerebral arteries, each point represents a pooled artery sample from three pups of the same dam. Bars represent mean ± SEM. Statistical comparisons were made using Kruskal–Wallis test with Dunn’s multiple comparisons test, with significance set at *p* < 0.05. * *p* < 0.05 by Dunn’s post hoc test. ** *p* < 0.01 by Dunn’s post hoc test. *** *p* < 0.001 by Dunn’s post hoc test.

Given that developing cerebral arteries produced measurable acetaldehyde following ex vivo alcohol exposure, we next examined whether these tissues express genes that encode key enzymes involved in alcohol metabolism. To validate the presence of genes encoding alcohol-metabolizing enzymes, we quantified mRNA expression of *Adh1*, *Cyp2e1*, and *Cat*—genes encoding alcohol dehydrogenase (ADH1), cytochrome P450 2E1 (CYP2E1), and catalase (CAT), respectively—in developing liver, brain, and cerebral artery tissue samples from alcohol-naïve PND 10 mouse offspring (Figure 2). Expression levels were normalized to *Gapdh* and are presented as 2^−ΔCt^ values on a logarithmic scale to allow detection of low-abundance transcripts.

Kruskal–Wallis tests were performed separately for each transcript to assess tissue-dependent differences in expression within each sex. In males, *Adh1* expression differed significantly across tissues (*H*(2) = 9.09, *p* = 0.011; Figure 2A left). Median expression levels were highest in liver (278.49, *n* = 7), followed by cerebral arteries (11.10, *n* = 7) and brain (0.040, *n* = 7). Post hoc Dunn’s multiple comparisons test revealed significantly greater *Adh1* expression in the liver compared with the brain (*p* = 0.0026), whereas differences between the liver and cerebral arteries and between the brain and cerebral arteries were not significant (*p* > 0.05). *Cyp2e1* expression also varied by tissue (*H*(2) = 11.38, *p* = 0.0034), with median expression following a pattern similar to *Adh1*—highest levels in the liver (561.20) and lowest in the brain (0.00075; Figure 2A middle). Post hoc Dunn’s test indicated significantly greater expression in the liver compared with the brain (*p* < 0.001), while expression in cerebral arteries was not different compared to the brain (*p* = 0.053). *Cat* expression did not differ significantly across tissues (*H*(2) = 4.64, *p* = 0.098); however, exploratory Dunn’s post hoc analysis identified significantly higher expression in the liver compared with the brain (*p* = 0.031; Figure 2A right).

In females, *Adh1* expression also differed significantly across tissues (*H*(2) = 6.82, *p* = 0.033), with median expression levels highest in the liver (228.32, *n* = 6) and lowest in the brain (0.19, *n* = 7; Figure 2B left). Dunn’s post hoc test showed significantly greater expression in the liver compared with the brain (*p* = 0.0094). Similar to the males, *Cyp2e1* expression varied significantly among tissues (*H*(2) = 9.76, *p* = 0.0076; Figure 2B middle). Post hoc Dunn’s test indicated significantly higher expression in the liver compared with the brain (*p* = 0.0018), while expression in cerebral arteries was not different compared to the brain (*p* = 0.095; Figure 2B middle). *Cat* expression did not differ significantly across tissues (*H*(2) = 3.10, *p* = 0.21) (Figure 2B right).

Collectively, these results confirm that developing cerebral arteries contain transcripts for the major alcohol-metabolizing enzymes *Adh1*, *Cyp2e1*, and *Cat*. While *Adh1* and *Cyp2e1* were expressed predominantly in the liver, the detection of *Cat* across all tissues—including developing cerebral arteries—supports the presence of this enzymatic pathway capable of contributing to local acetaldehyde production within the cerebrovasculature during early postnatal development.

### 2.3. Catalase Protein Is Detected in Developing Cerebral Arteries

**Figure 3:**
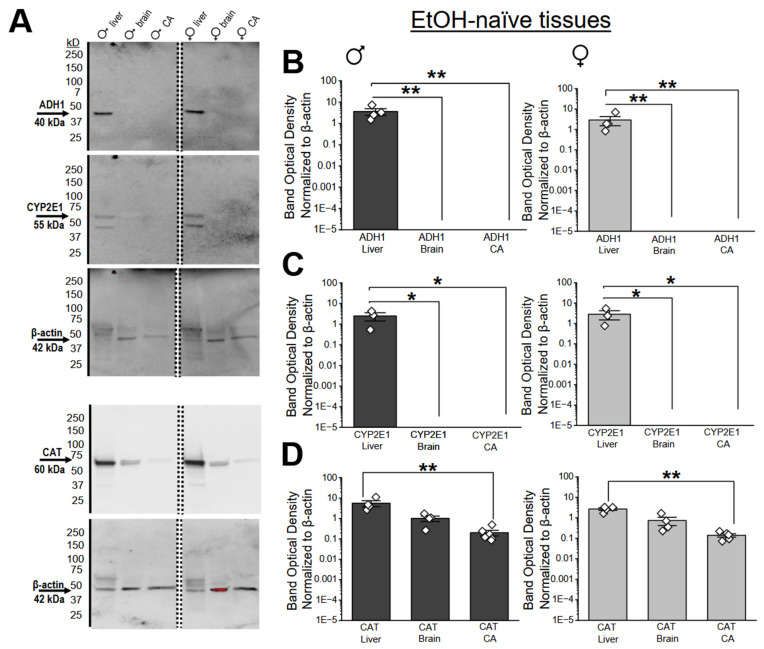
Western blot detection of alcohol-metabolizing enzymes in alcohol-naïve developing tissues. (**A**) Representative Western blots showing ADH1 (alcohol dehydrogenase; MW = 40 kDa), CYP2E1 (cytochrome P450 2E1; MW = 55 kDa), their β-actin (MW = 42 kDa) protein bands and CAT (catalase; MW = 60 kDa) with corresponding β-actin protein bands in developing liver, brain, and cerebral arteries (CA) from PND 10 mouse male and female offspring. Tissues probed: male liver, male brain, male CA, female liver, female brain, and female CA. (**B**) Quantification of protein band optical density (normalized to β-actin) for ADH1. Each point represents an individual pup for liver (male *n* = 4 dams; female *n* = 4 dams) and brain (male *n* = 4 dams; female *n* = 4 dams) tissue samples; each pup within a given sex was harvested from a separate dam. For CA, each data point represents data collected from a pooled artery sample of three pups of the same sex from a single dam (male *n* = 4 dams; female *n* = 4 dams). Here, and in (**C**,**D**), bars represent mean ± SEM. Males are represented by dark grey, and females are represented by light grey. Statistical comparisons were assessed using Kruskal–Wallis tests followed by Dunn’s multiple comparisons test. Significance was set at *p* < 0.05. ** *p* < 0.01 by Dunn’s post hoc test. (**C**) Quantification of protein band optical density (normalized to β-actin) for CYP2E1. Each point represents an individual pup for liver (male *n* = 3 dams; female *n* = 3 dams) and brain (male *n* = 3 dams; female *n* = 3 dams). Each pup within a given sex was harvested from a separate dam. For CA, each point represents data collected from a pooled artery sample from three pups of a single dam (male *n* =3 dams; female *n* = 3 dams). * *p* < 0.05 by Dunn’s post hoc test. (**D**) Quantification of protein band optical density (normalized to β-actin) for CAT. Each point represents an individual pup for liver (male *n* = 4 dams; female *n* = 4 dams) and brain (male *n* = 4 dams; female *n* = 4 dams). Each pup within a given sex was harvested from a separate dam. For CA, each point represents data collected from a pooled artery sample from three pups from the same dam (male *n* = 6 dams; female *n* = 5 dams). Outliers were removed prior to statistical analysis (female CAT liver *n* = 1; female CAT CA *n*= 1). Sex differences within each tissue were assessed using Mann–Whitney U tests and were not statistically significant for any protein or tissue (all *p* > 0.05).

Although qPCR confirmed transcription of *Adh1*, *Cyp2e1*, and *Cat* genes in developing tissues (Figure 2), we sought to determine whether transcriptional availability corresponded to detectable protein expression. Therefore, we performed Western blot analysis on liver, brain, and cerebral arteries from PND 10 alcohol-naïve offspring, and densities of ADH1-, CYP2E1-, and CAT-corresponding bands were quantified and normalized to β-actin (Figure 3). Protein densities are presented as 2^−ΔCt^ values on a logarithmic scale to allow visualization of lower abundance signals.

Consistent with transcript patterns showing predominant expression in the liver, ADH1 and CYP2E1 proteins were only detected in the liver (Figure 3A ADH1 and CYP2E1 representative blots). No significant sex differences were observed for liver ADH (males: 3.63 ± 1.27; females 2.89 ± 1.38; Mann–Whitney U = 5, *p* = 0.49) or CYP2E1 (males: 2.49 ± 1.08; females: 2.84 ± 1.35; Mann–Whitney U = 4, *p* = 1.0). Within each sex, tissue-dependent differences in protein expression were assessed using Kruskal–Wallis tests followed by Dunn’s post hoc comparisons. ADH1 protein levels differed significantly across tissues (*H*(2) = 10.46, *p* = 0.0054), with liver levels significantly higher than both brain and cerebral arteries in males and females (all *p* = 0.0051; Figure 3B). Similarly, CYP2E1 protein levels were significantly higher in the liver compared with the brain and cerebral arteries in both males and females (all *p* = 0.017; Figure 3C).

CAT protein was detected in all tissues, with the highest expression in the liver (males: 5.62 ± 1.88; females: 2.68 ± 0.39), intermediate in the brain (males: 1.01 ± 0.30; females: 0.74 ± 0.32), and lowest in the cerebral arteries (males: 0.20 ± 0.06; females: 0.14 ± 0.03; Figure 3A CAT representative blot). Within-sex Kruskal–Wallis analysis revealed significant tissue-dependent differences (males: *H*(2) = 10.88, *p* = 0.0043; females: *H*(2) = 10.19, *p* = 0.0061), and Dunn’s post hoc test showed that liver CAT protein was significantly higher than the cerebral arteries in both males and females (male: *p* = 0.0011; female: *p* = 0.0016). Brain CAT levels were not different compared with cerebral arteries (male: *p* = 0.085; female: *p* = 0.069); liver versus brain did not differ significantly as well (male: *p* = 0.15; female: *p* = 0.20; Figure 3D). Sex comparisons within each tissue were performed using Mann–Whitney U tests, which showed no significant differences in CAT protein expression between males and females in liver, brain, or cerebral arteries (all *p* > 0.05).

These results indicate that ADH1 and CYP2E1 protein expression are liver specific, whereas CAT is broadly expressed across all tissues, with graded abundance from the liver to cerebral arteries. Importantly, the broad expression of CAT, combined with the absence of detectable ADH1 or CYP2E1 protein in the cerebral arteries, suggests that CAT may serve as the main enzyme responsible for converting alcohol to acetaldehyde in cerebral arteries at this developmental stage.

### 2.4. CAT Inhibition Alters Acetaldehyde Production in Developing Cerebral Arteries

**Figure 4:**
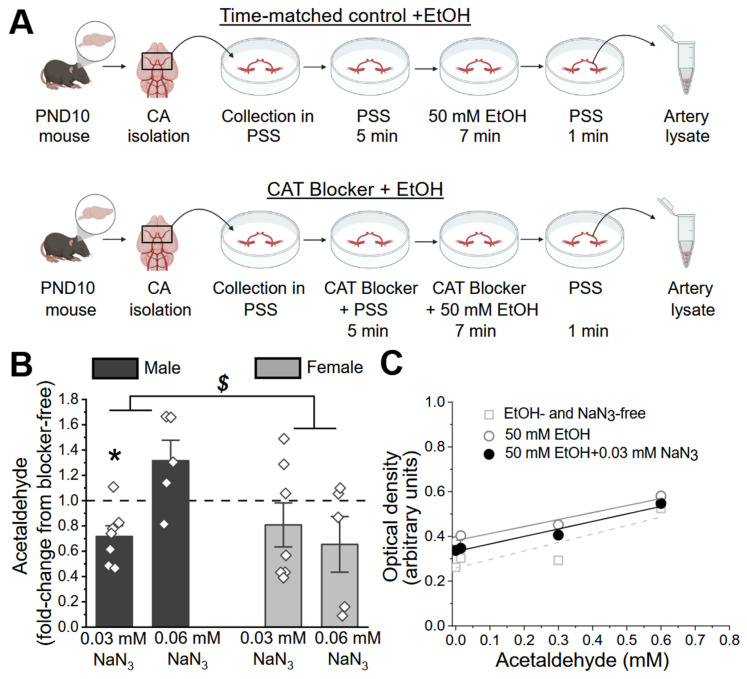
Acetaldehyde levels in the presence of a CAT enzyme blocker in developing cerebral arteries. (**A**) Experimental design: Isolated cerebral arteries from PND 10 mouse offspring were first incubated in PSS, then incubated for 5 min in either fresh PSS (time-matched control group) or CAT blocker-containing solution. Following incubation, arteries were then incubated in either alcohol (EtOH; 50 mM) or CAT blocker (sodium azide) and alcohol (EtOH; 50 mM) solution for 7 min. Afterwards, arteries were briefly dipped into PSS to remove excess alcohol (and blocker, if applicable) and immediately lysed for acetaldehyde quantification. (**B**) Acetaldehyde levels (expressed as fold-change relative to acetaldehyde levels detected following alcohol probing of arteries in time-matched controls). Data from developing cerebral arteries of male pups are in dark grey, and female pups are in light grey. CAT blocker, sodium azide (NaN_3_), was probed at 0.03 mM or 0.06 mM. Each point represents data collected from a pooled artery sample from three pups from the same dam (male 0.03 mM NaN_3_ *n* = 7 dams, male 0.06 mM NaN_3_ *n* = 5 dams; female 0.03 mM NaN_3_ *n* = 7 dams, female 0.06 mM NaN_3_ *n* = 5 dams). Time-matched control-incubated values used for normalization were obtained from male (*n* = 10 dams) and female (*n* = 11 dams) pooled artery samples. Data are presented as mean ± SEM. Statistical comparisons were made using two-way ANOVA, with significance set at *p* < 0.05. $ *p* < 0.05 by two-way ANOVA (interaction between blocker concentration and sex). * *p* < 0.05 by Mann–Whitney U test (significant difference compared to time-matched control levels). (**C**) Optical density as a function of acetaldehyde concentration in acetaldehyde detection kit standards that included alcohol (EtOH; 50 mM) or alcohol mixture with sodium azide (50 mM EtOH + 0.03 mM NaN_3_). Linear fits were obtained using the built-in function in the Origin 2023 software (OriginLab).

Because CAT was the only alcohol-metabolizing enzyme detected at the protein level in developing cerebral arteries, we next tested whether blocking CAT activity affects alcohol-derived acetaldehyde formation. For this, cerebral arteries were harvested from PND 10 offspring and incubated with differing concentrations of a selective CAT inhibitor, sodium azide (NaN_3_; 0.03 mM and 0.06 mM; Figure 4A) [34,35]. These concentrations were intentionally chosen to achieve selective modulation of catalase activity under conditions that preserve arterial viability. Incubation of arteries and quantification of acetaldehyde levels was conducted as described in Methods. Acetaldehyde levels were normalized to their respective means of sex-matched time-matched controls.

A two-way ANOVA (factors: blocker concentration and sex) revealed no significant main effect of blocker concentration (F(1, 20) = 1.89, *p* = 0.18) or sex (F(1, 20) = 1.94, *p* = 0.18). However, there was a significant interaction between blocker concentration and sex (F(1, 20) = 5.41, *p* < 0.05), indicating that the effect of NaN_3_ on acetaldehyde production differs between males and females. Therefore, responses were examined separately by sex.

At the lower blocker concentration (0.03 mM NaN_3_), acetaldehyde production was reduced relative to time-matched controls in both males and females (Figure 4B). Male cerebral arteries produced 0.72 ± 0.08-fold acetaldehyde relative to their levels in the presence of 50 mM alcohol without CAT blocker (1.27 × 10^−5^ ± 1.62 × 10^−6^ mg/µg of total protein). Similarly, female cerebral arteries displayed a 0.81 ± 0.17-fold acetaldehyde production relative to the alcohol-only controls (1.48 × 10^−5^ ± 1.60 × 10^−6^ mg/µg of total protein). This reduction in acetaldehyde production was not a result of sodium azide interference with acetaldehyde detection kit components, as standard readings did not get modified by the addition of sodium azide into the kit’s working reagent (Figure 4C).

In contrast, at the higher concentration (0.06 mM NaN_3_), the response differed by sex (Figure 4B). Male arteries showed an increase in acetaldehyde production (1.32 ± 0.16-fold relative to time-matched controls), whereas female arteries exhibited a decrease (0.65 ± 0.22-fold). The corresponding time-matched control levels were 1.15 × 10^−5^ ± 4.70 × 10^−6^ mg/µg of total protein in males, and 3.28 × 10^−5^ ± 1.72 × 10^−5^ mg/µg of total protein in females.

Taken together, these findings demonstrate that CAT inhibition alters acetaldehyde production in developing cerebral arteries ex vivo, further indicating that CAT activity defines local acetaldehyde generation.

## 3. Discussion

In this study, we examined whether developing cerebral arteries are capable of metabolizing alcohol into acetaldehyde, a reactive and toxic metabolite strongly implicated in alcohol-induced tissue injury [36,37,38,39]. Using ex vivo alcohol incubation in PND 10 (third trimester-equivalent of human pregnancy) mouse offspring, we quantified acetaldehyde production, assessed transcript and protein expression of the three major alcohol-metabolizing enzymes (ADH1, CYP2E1, and CAT), and tested whether pharmacological inhibition of CAT blunted acetaldehyde formation ex vivo. The chosen developmental stage corresponds to the end of the third trimester-equivalent of human pregnancy as determined by comparing developmental milestones between the rodent and human brain [33]. Evaluation of the cerebral artery function during the embryonic stage may not be feasible as mouse embryos are extremely small and more importantly, the cerebral circulation is only morphologically established by the end of the first trimester [40]. Although alcohol metabolism could potentially be evaluated during the second trimester-equivalent of human pregnancy in mice (gestational days 11–21), our recent work identified the third trimester-equivalent as the most vulnerable period to alcohol exposure based on assessment of oxygen consumption and respiratory parameters of the mitochondria [41]. Our overarching hypothesis in the present work was that developing cerebral arteries possessed intrinsic metabolic capacity, enabling local acetaldehyde generation. To test this, we isolated developing cerebral arteries and used selective pharmacological inhibition to determine CAT’s functional contribution to alcohol metabolization. Collectively, our findings indicate that the developing cerebrovasculature may actively participate in alcohol bioactivation during a critical window of brain development.

Developmental alcohol exposure is the sole cause of FASD, a spectrum of disorders characterized by persistent cognitive, behavioral, and physical impairments [1,2,3,4]. Exposure during third trimester of pregnancy has been linked to growth deficits and specific motor and cognitive abnormalities [32]. Cerebral circulation is essential for early brain development, supporting neurovascular coupling, nutrient delivery, angiogenesis, and waste clearance [13,14,15,16,17]. PAE disrupts these processes, impairing cerebral blood flow, altering vascular density, and compromising endothelial and smooth muscle function across species [11,42,43]. Despite the developing cerebrovasculature being a recognized target of PAE, the mechanisms by which alcohol directly affect immature vessels remain poorly defined. Local metabolism of alcohol to acetaldehyde within vascular tissue is one plausible but understudied mechanism, with potential to increase oxidative stress, alter vascular tone, and impair endothelial signaling [26,44,45,46,47]. Our study paves the way to address this gap by examining whether alcohol metabolization into acetaldehyde may take place in developing cerebral arteries.

Our first major finding is that developing cerebral arteries in an ex vivo setting produce measurable acetaldehyde even without the exogenous challenge with alcohol. Acetaldehyde is produced in minimal amounts during natural metabolic processes, and its rapid utilization mitigates toxicity [48,49,50]. During ex vivo alcohol exposure to mild (13 mM) and moderate-to-heavy (50 mM) alcohol concentrations, acetaldehyde production is boosted beyond baseline levels (Figure 1D). These concentrations correspond to approximately 0.06% and 0.23% blood alcohol levels, respectively; 13 mM EtOH is near, but below, the legal limit of intoxication to drive a motor vehicle (0.08% blood alcohol) while 50 mM EtOH reflects blood levels during heavy intoxication. Acetaldehyde production during this exposure type demonstrates the intrinsic metabolic capacity of developing cerebral arteries. Although hepatic metabolism is responsible for most systemic acetaldehyde production, extrahepatic tissues—including adult brain, pancreas, and vasculature—also express alcohol-metabolizing enzymes at low-to-moderate levels to enable natural metabolic pathways [44,51,52,53,54,55]. Importantly, developmental expression patterns of alcohol-metabolizing enzymes differ from adults. For example, neonatal liver shows limited ADH activity or mixed enzymatic contributions [56,57]. Our observations provide the first evidence that alcohol metabolism may occur directly within the early cerebrovasculature—a period marked by rapid maturation of vascular structure, myogenic tone formation, and establishment of neurovascular coupling in parenchymal vessels and microvasculature [21,58]. As we only tested developing cerebral arteries during a single PND 10 timepoint and only ex vivo, the window of vulnerability or emergence of alcohol-metabolizing capacity within the developing cerebrovasculature in vivo remains to be established.

Second, we found that genes encoding all three major alcohol-metabolizing enzymes (*Adh1*, *Cyp2e1*, and *Cat*) are expressed in alcohol-naïve developing cerebral arteries, with relative expression levels lower than those in the liver (Figure 2A,B). These tissue-specific differences parallel known developmental trajectories, wherein hepatic enzyme expression undergoes robust postnatal maturation, while brain expression remains regionally restricted [28,59,60]. High expression of *Adh1*, *Cyp2e1*, and *Cat*-coding genes in the liver reflects the liver’s dominant role in alcohol metabolism, and the comparatively limited metabolic capacity of neural and vascular tissues [27,28]. Our data extend this framework by showing that developing cerebral arteries themselves express genes encoding these enzymes, indicating that vascular cells contain the molecular machinery required for alcohol oxidation. This raises the possibility that local metabolism may contribute to cerebrovascular oxidative stress and signaling disruptions observed in PAE models [61,62].

Third, protein-level analysis revealed that catalase (CAT) was the alcohol-metabolizing enzyme robustly detected in developing cerebral arteries, while ADH1 and CYP2E1 proteins were below detection limits (Figure 3A–D). This pattern aligns with developmental metabolic biology: catalase is highly expressed in neonatal tissues, particularly before hepatic ADH fully matures [56,63], and catalase drives the majority of alcohol oxidation in neonatal rodent whole-brain homogenates [64,65]. Our results further extend these findings by demonstrating that developing cerebral arteries themselves contain sufficient CAT protein to metabolize alcohol, suggesting that CAT-dependent acetaldehyde generation may contribute to alcohol-driven pathology of cerebrovasculature during development. The cellular location of CAT within blood vessel tissue remains elusive. In adult cerebral arteries, the majority of cellular content is presented by smooth muscle cells [66]. They differentiate from endothelial cell precursors [67]. Our laboratory has recently documented that in primates, cerebrovascular smooth muscle differentiation emerges gradually, with the inner cerebral artery layer and remaining wall components having similar expression levels of endothelial and smooth muscle markers at the end of the second trimester-equivalent of human pregnancy [41]. By birth, smooth muscle differentiation may still be incomplete. This is because myogenic tone and ability to autoregulate—key features of cerebral arteries driven by smooth muscle differentiation—are reported only to be established by the end of the third trimester-equivalent (perinatal period in humans) with increased vulnerability lasting into postnatal development [68]. Thus, there may not be a clear distinction between key cerebrovascular cellular phenotypes during early development.

Finally, functional inhibition experiments focused exclusively on CAT, as it was the only alcohol-metabolizing enzyme that we were able to detect at the protein level in PND 10 mouse cerebral arteries. Sodium azide, a CAT inhibitor [34], did not produce a clear dose-dependent suppression of acetaldehyde formation at either concentration (0.03 or 0.06 mM). Although acetaldehyde production was modestly reduced at 0.03 mM, the overall reduction was not statistically significant between the two doses (Figure 4B). Unexpectedly, the higher sodium azide concentration (0.06 mM) did not further suppress acetaldehyde production and instead produced a sex-divergent response—acetaldehyde increased in males but decreased in females (Figure 4B). These variable outcomes raise questions regarding tissue viability and probability of off-target effects of sodium azide. The concentrations used in this study have been reported to selectively modulate CAT activity while maintaining tissue viability and minimizing reported off-target metabolic disruptions largely associated with sodium azide targeting of heme group-containing proteins [69,70,71]. Indeed, sodium azide could effectively inhibit complex IV within the mitochondrial respiratory chain, thus, potentially limiting tissue viability. However, inhibition of complex IV requires much higher concentrations of sodium azide, approximately 10 folds greater than ones used in the present study [72]. Thus, this pathway is unlikely to drive observed changes in alcohol metabolism. Moreover, while sodium azide does not target catalase exclusively but rather binds to heme groups within a multitude of proteins, in our study, this blocker was used as a verification step after Western blot analysis clearly showed no detectable bands corresponding to alcohol dehydrogenase and CYP2E1 in cerebral artery lysates from mouse pups (Figure 2). Alcohol dehydrogenase does not contain a heme group but rather relies on a zinc catalytic center [73], making it an unlikely off-target of sodium azide. While CYP2E1 contains a heme group [74], the lack of detectable CYP2E1 protein on Western blot argues against major involvement of this enzyme into observed alcohol metabolism within developing cerebral arteries. Together, these findings indicate that CAT largely contributes to acetaldehyde generation in developing cerebral arteries. Inhibition of this enzyme reveals a sexually dimorphic metabolic plasticity that enables a sex-dependent increase in acetaldehyde production in males when CAT is blocked by high concentrations of sodium azide. Potential explanations include male-specific off-target effects of sodium azide, sex-dependent enzyme de-sensitization to its blocker, or the presence of alternative or compensatory pathways that induce other alcohol-metabolizing enzymes.

Overall, our ex vivo findings demonstrate that developing cerebral arteries possess both the molecular and functional capacity to metabolize alcohol and produce acetaldehyde. These findings may have important implications for understanding cerebrovascular vulnerability during PAE. Local acetaldehyde generation may enhance oxidative stress, disrupt calcium signaling, and impair vasoactive responses—processes known to be disrupted in PAE models [61,62,75]. The presence of CAT protein and acetaldehyde production in the absence of detectable ADH1 or CYP2E1 proteins further indicates that neonatal cerebrovascular alcohol metabolism differs fundamentally from adult patterns, relying heavily on CAT during this developmental period when vascular maturation is especially sensitive to metabolic and oxidative cues. Whether CAT-driven alcohol metabolization into acetaldehyde within developing cerebral arteries in vivo could contribute to early-life cerebrovascular vulnerability to alcohol insult and eventually, to FASD pathogenesis, remains to be determined.

Several limitations warrant consideration. First, the ex vivo design excludes systemic contributions such as circulating alcohol, maternal metabolites, or hepatic clearance, all of which likely influence in vivo acetaldehyde dynamics. The short incubation period captures only acute metabolic events and may not reflect longer-term compensatory responses. Second, the variability observed in qPCR datasets may be attributable, in part, to the use of gross cortical tissue collections rather than the precise microdissection of distinct subregions. This approach may have introduced heterogeneity arising from region-specific developmental differences at PND 10. Additionally, the variability and occasional bimodal distributions observed in qPCR results may reflect a combination of technical and biological factors. Third, protein analyses were limited to ADH1, CYP2E1, and CAT, and therefore, contributions from additional ADH and CYP isoforms cannot be excluded. Western blot sensitivity may have limited detection of very low abundance of ADH1 or CYP2E1 proteins that could still contribute functionally to alcohol metabolism. Fourth, sodium azide, while widely used as a catalase inhibitor, lacks complete specificity and may produce off-target effects, underscoring the need for follow-up studies using alternative inhibitors or direct enzymatic activity assays. Finally, our study focused on PND 10, a single developmental timepoint; given that cerebrovascular enzyme expression likely evolves across development, future studies should characterize alcohol-metabolizing capacity from prenatal stages through adolescence.

Despite these limitations, our study provides the first direct evidence that developing cerebral arteries may metabolize alcohol to acetaldehyde, express key alcohol-metabolizing genes, and rely predominantly on catalase protein for metabolic activity at this developmental stage. Our ex vivo findings point at a previously unrecognized source of acetaldehyde within the developing brain and highlight catalase-mediated alcohol metabolism as a potential contributor to cerebrovascular dysfunction associated with PAE.

## 4. Materials and Methods

### 4.1. Subjects

The study was conducted using C57BL/6J mice purchased from The Jackson Laboratory. Upon arrival at the University of Tennessee Health Science Center campus, animals were acclimated for no less than 3 days. Nulliparous female mice, 8- to 9-weeks old, were single-pair mated with male mice, 10- to 11-weeks old, in-house. On the day of the experiment, approximately 10 ± 1 days after parturition, dams and their offspring were deeply anesthetized with isoflurane using the “drop in a jar” method and when toe-pinch sensitivity was lost, animals were decapitated with sharp scissors.

For cerebral artery analyses, the middle cerebral artery (MCA) and anterior cerebral artery (ACA) were dissected from all animals to ensure comparable vessel segments between litters. Cerebral arteries from three pups of the same sex from the same dam were pooled to constitute one data point (*n*). Male and female samples were processed separately.

### 4.2. Study with Animals and Ethics Statement

All animals were housed and handled according to the institutional animal care requirements of the University of Tennessee Health Science Center. All experimental procedures were conducted under an approved Institutional Animal Care and Use Committee protocol.

### 4.3. Determination of Acetaldehyde Levels in Developing Cerebral Arteries Ex Vivo

Immediately after mouse decapitation, offspring cerebral arteries were dissected out of brain tissue under a microscope and placed into a plate filled with physiological saline solution (PSS) of the following composition (mM): 119 NaCl, 4.7 KCl, 1.2 KH_2_PO_4_, 1.2 MgSO_4_, 0.023 EDTA, 11 C_6_H_12_O_6_ (glucose), 24 NaHCO_3_, and 1.6 CaCl_2_. Arteries were carefully cleaned of surrounding pia mater and then randomized into the following groups: PSS versus alcohol, and time-matched control versus alcohol in presence of CAT blocker.

*PSS versus alcohol.* Isolated cerebral arteries in the alcohol exposure group were initially kept in PSS prior to a 7 min long incubation with alcohol (13 mM or 50 mM ethyl alcohol, EtOH). Seven minutes of incubation in alcohol were chosen because alcohol administration has an acute effect on the cerebral artery diameter in adult mice within 5–10 min [76]. Following incubation with alcohol, arteries were dipped into PSS and immediately lysed with RIPA buffer (Thermo Scientific, Rockford, IL, USA) in the presence of a Halt Protease Inhibitor Cocktail (Thermo Scientific, Rockford, IL, USA). Following five 5 s long sonication episodes on ice using the low-intensity setting on the Sonic Dismembrator Model 100 (Fisher Scientific), lysate was centrifuged at 14,000 rpm at 4 °C for 5 min until clear separation of layers was obtained. Supernatants were collected, and the amount of total protein per 1 mL of supernatant was quantified using the Pierce BCA Protein Assay Kit (Thermo Scientific, Rockford, IL, USA). Acetaldehyde levels in the supernatant were measured using the Acetaldehyde Assay Kit (MAK434 by Sigma-Aldrich, St. Louis, MO, USA) according to the manufacturer’s protocol with declared detection between 2 μM and 2 mM. Acetaldehyde content (mg) was normalized to total protein content (µg) and expressed as fold-change relative to the mean value of the time-matched control group (PSS, no alcohol exposure).

### 4.4. Alcohol Exposure in the Presence of Time-Matched Control Versus Alcohol Metabolism Blocker of CAT

For enzyme inhibition studies, isolated cerebral arteries were first maintained in PSS, followed by a 5 min preincubation with differing concentrations of sodium azide (0.03 mM or 0.06 mM; NaN_3_) with the stock solution being diluted in Milli-Q water [77]. Arteries were then incubated in PSS containing 50 mM alcohol in the continued presence of the blocker for an additional 7 min. Following incubation, arteries were dipped into PSS and underwent protein and acetaldehyde quantifications as described above for PSS versus alcohol groups. Time-matched control arteries underwent the same protocol: initial maintenance in PSS, 5 min preincubation in vehicle, 7 min long incubation in PSS containing 50 mM alcohol and continued the presence of vehicle, and a final wash in PSS. For each sodium azide concentration tested, time-matched solutions were prepared by adding the corresponding volume of solvent (Milli-Q water) to 30 mL PSS to match the final dilutions of sodium azide.

### 4.5. Determination of Gene Expression in Developing Tissues

Immediately following mouse decapitation, developing tissues of interest from offspring were harvested for quantitative PCR (qPCR) analysis. Approximately 5 mg each of liver, cerebrum (for regional consistency, samples were collected from frontal cortical tissue via the inferior view of the brain), and cerebral arteries was collected and immediately frozen at −80 °C until used for RNA isolation. Total RNA was isolated from all tissue samples using the RNeasy Plus Micro Kit (Qiagen, Hilden, Germany) and quantified with a NanoDrop One Microvolume UV–Vis Spectrophotometer (Thermo Scientific, Madison, WI, USA). Isolated RNA was stored at −80 °C. Reverse transcription was performed using the High-Capacity cDNA Reverse Transcription Kit (Applied Biosystems, Vilnius, Lithunia) on a ProFlex PCR system (Applied Biosystems) to generate complementary DNA (cDNA). Subsequent qPCR was carried out on QuantStudio5 (Applied Biosytems) with TaqMan Fast Advanced Master Mix and TaqMan gene expression assays for *Adh1* (Mm00507711_m1), *Cyp2e1* (Mm00491127_m1), *Cat* (Mm00437992_m1), and *Gapdh* (Mm99999915_g1) (ThermoFisher Scientific, Pleasanton, CA, USA). The latter was used as the control. All procedures were performed in accordance with the manufacturer’s instructions. Relative gene expression was calculated using the 2^−ΔCt^ method following published guidelines [78].

### 4.6. Detection of Alcohol Metabolizing Enzyme Proteins in Developing Tissues Using Western Blot

Tissues of interest were immediately collected after mouse decapitation. Samples included developing liver, cerebrum, and cerebral arteries from offspring. All tissue samples were lysed with RIPA buffer (Thermo Scientific, Rockford, IL, USA) in the presence of Halt Protease Inhibitor Cocktail (Thermo Scientific, Rockford, IL, USA). Lysates were sonicated five times on ice, each sonication lasting for 5 s, and then centrifuged at 14,000 rpm at 4 °C for 15 min until clear separation of layers was achieved. Supernatants were collected and stored at −80 °C. Concentration of total protein within each lysate was determined using the Pierce BCA Protein Assay Kit (Thermo Scientific), Rockford, IL, USA. Then, lysates were diluted with nuclease-free water according to protein quantification and loaded into 4–20% Mini-PROTEAN TGX Gels (Bio-Rad, Hercules, CA, USA) at 30 µg total protein per well. Electrophoresis was performed at 90 V for 90 min, followed by transfer of proteins onto Nitrocellulose Membranes (Bio-Rad, Hercules, CA, USA).

Western blot analyses were performed using the same membrane(s) per experimental run, with sequential cycles of antibody probing, imaging, stripping, and re-probing. For each target protein, membranes were incubated with the appropriate primary and secondary antibodies, imaged, and then stripped prior to incubation with the next antibody (see below for details). β-actin was consistently used as the loading control and was detected on the same membranes as target proteins. Primary antibodies included alcohol dehydrogenase (ADH1; rabbit monoclonal, ab108203, 1:1000 dilution), cytochrome P450 2E1 (CYP2E1; rabbit polyclonal, ab28146, 1:1000 dilution), catalase (CAT; rabbit monoclonal, ab209211, 1:2000 dilution), and β-actin (mouse monoclonal, ab8226, 1:5000 dilution). All primary antibodies were purchased from Abcam.

Membranes were blocked for 1 h shaking at room temperature in the appropriate primary antibody buffer, either 5% non-fat milk or 5% bovine serum albumin (BSA; depending on primary antibody of interest). Each membrane was incubated overnight at 4 °C shaking in the dark with one primary antibody at a time. Antibodies with rabbits as a host species were diluted in 5% non-fat milk, while β-actin was diluted with 5% BSA.

The following morning, after primary antibody washout with TBST, membranes that were probed for ADH1, CYP2E1, or CAT were incubated with goat anti-rabbit IgG whole antibodies conjugated with HRP at 1:5000 dilution (goat polyclonal, 65-6120, Invitrogen) for 1 h shaking at room temperature, then washed three times with TBST. Membranes probed for β-actin were incubated with goat anti-mouse IgG whole antibodies conjugated with HRP at 1:5000 dilution (goat polyclonal, 62-6520, Invitrogen) under the same conditions and then washed three times with TBST.

In an amount of 2 mL, SuperSignal West Femto Maximum Sensitivity Substrate (Thermo Scientific) was added to each membrane before blot images were developed using the “Chemiluminescence” setting in the Quantity One software on the Universal Hood machine (Bio-Rad). Images were obtained after an 8 min long manual exposure. Relative quantification of protein bands from Western Blot was performed with the free software, ImageJ (version 1.46r), following published instructions.

### 4.7. Chemicals

Sodium azide (NaN_3_) was purchased from Sigma-Aldrich. Ethyl alcohol (190 proof) was purchased from Thermo Scientific. On the day of the experiment, sodium azide was dissolved in Milli-Q water to prepare a stock solution of 1 mM, and then further diluted with PSS to the desired final concentrations. Alcohol was directly added in PSS to the desired final concentrations.

### 4.8. Statistical Analysis and Data Representation

Statistical analysis was performed using the free online resource, Statistics Kingdom. Statistical comparisons were conducted using Mann–Whitney U tests and Kruskal–Wallis tests followed by Dunn’s post hoc test, and two-way ANOVA test, depending on the data distribution (Gaussian versus non-Gaussian) and number of experimental groups. Sex was considered a biological variable throughout all analyses. For experiments involving experimental treatments, statistical comparisons were performed using two-way ANOVA with sex and treatment condition as factors to assess potential sex-dependent responses. In contrast, for analyses aimed at characterizing baseline tissue-specific expression patterns under alcohol-naïve conditions, males and females were analyzed separately to evaluate within-sex tissue differences. Where appropriate, sex comparisons within a given tissue were performed using Mann–Whitney U tests. The specific statistical methods used for each analysis are detailed in the legends. Statistical significance was defined as *p* < 0.05. Data are expressed as mean ± standard error of means (SEM). Each data point (“n”) within a specific tissue (e.g., offspring brain or liver) was obtained from a separate animal donor. To account for litter effects, no more than three data points of same sex from the same litter were included into a given experimental group. In the case of offspring cerebral arteries, each data point represents pooled arteries of three separate donors of the same sex from the same dam. In this case, “n” represents number of dams whose progeny was used. Final plotting and graphing were carried out in Origin 2023 software (Origin Lab). Outliers were excluded using Origin 2023’s built-in function based on Grubbs’ test and Dixon’s Q-test. Number of excluded values is specified in the legends.

## 5. Conclusions

In conclusion, our findings establish that developing cerebral arteries are capable of oxidizing alcohol to acetaldehyde when alcohol is probed at levels that are reached within blood circulation during mild and moderate-to-heavy alcohol consumption. To the best of our knowledge, this is the first study to demonstrate that the enzymatic components required for alcohol metabolism are present and functionally active within the developing cerebrovasculature, revealing the cerebral artery as a previously unrecognized site of fetal alcohol metabolism. These results broaden our understanding of how alcohol may impact cerebrovascular physiology and highlight the importance of considering local, tissue-specific alcohol metabolism when evaluating mechanisms of fetal brain vulnerability. Future studies will aim to determine how arterial alcohol metabolism influences oxidative stress, vascular reactivity, and long-term neurovascular outcomes associated with FASD.

## Data Availability

The raw data supporting the conclusions of this article will be made available by the authors on request.
